# EM23, A Natural Sesquiterpene Lactone from *Elephantopus mollis*, Induces Apoptosis in Human Myeloid Leukemia Cells through Thioredoxin- and Reactive Oxygen Species-Mediated Signaling Pathways

**DOI:** 10.3389/fphar.2016.00077

**Published:** 2016-03-29

**Authors:** Hongyu Li, Manmei Li, Guocai Wang, Fangyuan Shao, Wenbo Chen, Chao Xia, Sheng Wang, Yaolan Li, Guangxiong Zhou, Zhong Liu

**Affiliations:** ^1^Institute of Traditional Chinese Medicine and Natural Products, College of Pharmacy, Jinan UniversityGuangzhou, China; ^2^Faculty of Health Sciences, University of MacauMacau, China; ^3^Guangzhou Jinan Biomedicine Research and Development Center, Guangdong Provincial Key Laboratory of Bioengineering Medicine, College of Life Science and Technology, Jinan UniversityGuangzhou, China

**Keywords:** ROS, myeloid leukemia, apoptosis, thioredoxin, ASK1

## Abstract

*Elephantopus mollis* (EM) is a traditional herbal medicine with multiple pharmacological activities. However, the efficacy of EM in treating human leukemia is currently unknown. In the current study, we report that EM23, a natural sesquiterpene lactone isolated from EM, inhibits the proliferation of human chronic myeloid leukemia (CML) K562 cells and acute myeloid leukemia (AML) HL-60 cells by inducing apoptosis. Translocation of membrane-associated phospholipid phosphatidylserines, changes in cell morphology, activation of caspases, and cleavage of PARP were concomitant with this inhibition. The involvement of the mitochondrial pathway in EM23-mediated apoptosis was suggested by observed disruptions in mitochondrial membrane potential. Mechanistic studies indicated that EM23 caused a marked increase in the level of reactive oxygen species (ROS). Pretreatment with *N*-acetyl-L-cysteine, a ROS scavenger, almost fully reversed EM23-mediated apoptosis. In EM23-treated cells, the expression levels of thioredoxin (Trx) and thioredoxinreductase (TrxR), two components of the Trx system involved in maintaining cellular redox homeostasis, were significantly down-regulated. Concomitantly, Trx regulated the activation of apoptosis signal-regulating kinase 1 (ASK1) and its downstream regulatory targets, the p38, JNK, and ERK MAPKs. EM23-mediated activation of ASK1/MAPKs was significantly inhibited in the presence of NAC. Furthermore, tumor necrosis factor alpha (TNF-α)-mediated activation of nuclear factor-κB (NF-κB) was suppressed by EM23, as suggested by the observed blockage of p65 nuclear translocation, phosphorylation, and reversion of IκBα degradation following EM23 treatment. Taken together, these results provide important insights into the anticancer activities of the EM component EM23 against human CML K562 cells and AML HL-60 cells.

## Introduction

Leukemia, a malignant disease affecting blood cells, usually begins in the bone marrow and is characterized by an aberrant accumulation of abnormal white blood cells. Leukemia can be clinically and pathologically subdivided into four main types: acute lymphoblastic leukemia (ALL), acute myeloid leukemia (AML), chronic lymphocytic leukemia (CLL), and chronic myeloid leukemia (CML), as well as many less common types (Vardiman et al., 2009). The treatment of leukemia is complicated and is influenced by the age and overall health of an affected patient as well as by their type of leukemia, in addition to other factors. However, chemotherapy remains one of the major therapeutic approaches for leukemia.

Both AML and CML are myeloid or myelogenous leukemias, and the initial cancerous changes associated with these cancers affect certain blood-forming cells in the bone marrow. Currently, bone marrow transplantation/allogeneic stem cell transplantation is the only curative treatment for CML (Goldman et al., 2010). TKIs that target BCR-ABL are the standard treatment for CML. Imatinib mesylate, a first-generation TKI (Kantarjian et al., 2002), has produced excellent clinical results in terms of achieving high remission rates and improving prognosis (Hughes et al., 2006; Bhamidipati et al., 2013). New second- and third- generation TKIs, such as dasatinib, nilotinib, and bosutinib, exhibit superior inhibitory activity against BCR-ABL compared to imatinib. Although TKIs are extensively used, serious drug-drug interactions and emerging resistance of CML cells to TKIs through multiple mechanisms has limited their clinical application (Volpe et al., 2009).

AML is the most common acute leukemia in adult patients. This type of leukemia progresses rapidly and may be lethal within weeks or months. Based on the cytogenetic/genetic features of AML, and according to the 2008 revised WHO classification system, patients can be broadly classified into three different risk groups. Treatment strategies and prognoses vary among these subtypes and are further influenced by patient-specific factors (Grimwade et al., 2010; Zeisig et al., 2012). All AML subtypes, except for acute promyelocytic leukemia (APL), are typically treated with induction chemotherapy using a “3+7” combination of daunorubicin and cytarabine (Dohner et al., 2010; Zeisig et al., 2012). Treatment with all-trans retinoic acid, which can effectively induce APL cell differentiation, can achieve complete remission in almost all APL patients (Degos and Wang, 2001). Conversely, although treatment strategies for AML have substantially progressed in recent decades, effective therapies for non-APL subtypes of AML are still urgently needed.

Tumor cells usually have a more active metabolism than normal cells to maintain their rapid growth and escape from cell death (Hanahan and Weinberg, 2011). Leukemia cells undergo many pathological changes compared to normal blood cells. Therefore, the identification of phytocompounds from medicinal or edible plants that affect multiple intracellular targets may lead to the discovery of a novel, effective and multifunctionalanti-leukemia drug or prodrug.

*Elephantopus mollis* (EM) belongs to the Asteraceae family and is used as a folk medicine in China. EM possesses multiple pharmacological activities, including anticancer activity (Tabopda et al., 2008; Ooi et al., 2011, 2012), anti-protozoal activity (Gachet et al., 2010), melanogenesis inhibition activity (Hasegawa et al., 2010), bone regenerative activity (Ngueguim et al., 2012), and hepatoprotective activity (Lin et al., 1995). However, whether EM is an efficacious treatment option for human AML and CML remains unknown.

In this study, we investigated the anti-leukemia properties and associated molecular mechanisms of EM23, a natural sesquiterpene lactone isolated from EM, in the K562 and HL-60 human CML and AML cell lines. Mechanistically, we demonstrated that EM23 inhibited the mammalian Trx system, interfered with cellular redox homeostasis and resulted in ROS-dependent apoptosis by regulating complex signaling pathways, including those governed by ASK1, MAPK, and NF-κB.

## Materials and Methods

### Cell Culture and Reagents

The human CML cell line K562, the human APL cell line HL-60, human liver cell line HL-7702 and mouse embryonic fibroblast cell line NIH/3T3 (NIH/swiss) were obtained from the Cell Bank of the Chinese Academy of Sciences (Shanghai, China). K562 and HL-60 cells were cultured in RPMI 1640 medium (Life Technologies, Grand Island, NY, USA). HL-7702 and NIH/3T3 cells were grown in DMEM medium (Life Technologies, Grand Island, NY, USA). All cell lines were grown in specific media supplemented with 10% fetal bovine serum (FBS, Gibco), 100 U/mL penicillin and 100 μg/mL streptomycin (Invitrogen, Carlsbad, CA, USA). The cells were grown in a 5% CO_2_ humidified atmosphere in incubators maintained at 37°C.

**FIGURE 1:**
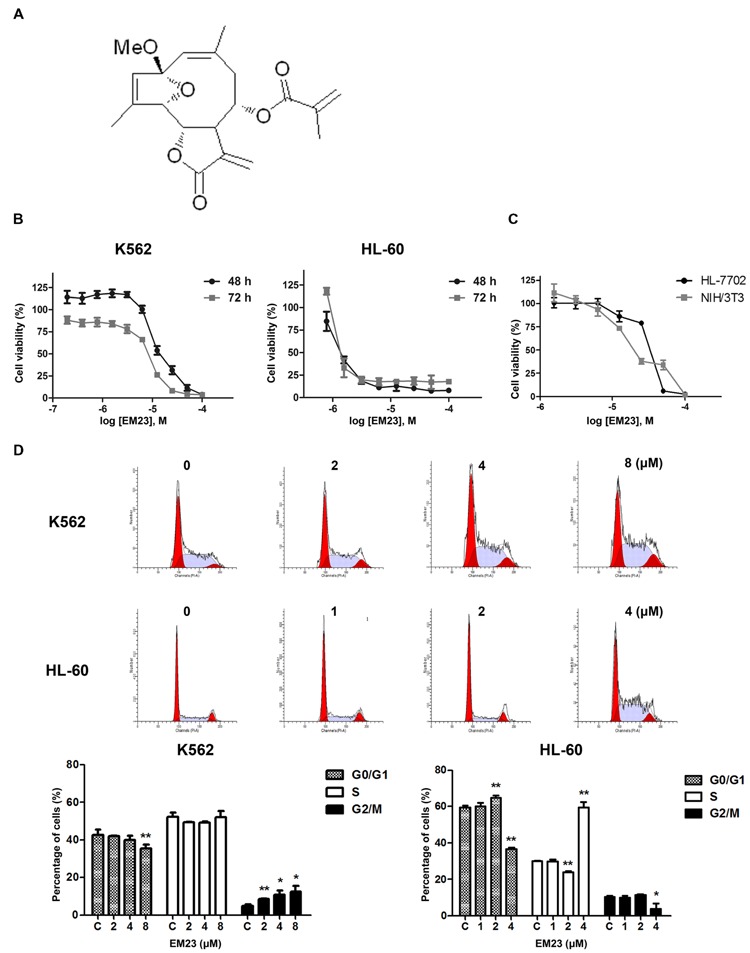
**EM23 inhibits cell proliferation and cell cycle progression. (A)** Chemical structure of EM23. **(B)** Effects of EM23 on human myeloid leukemia cell proliferation. K562 and HL-60 cells were treated with various concentrations of EM23 for 48 and 72 h, respectively. Cell viability was measured using a CCK-8 assay. **(C)** Cytotoxic effects of EM23 on normal mammalian cells. HL-7702 and NIH/3T3 cells were treated with various concentrations of EM23 for 48 h. Cell viability was measured using a CCK-8 assay. **(D)** Effects of EM23 on cell cycle distribution. Following treatment with EM23 for 48 h, cells were fixed and stained with PI solution. Cell cycle distribution was measured by flow cytometry. All of data are presented as the mean ± SD of at least three independent experiments. ^∗^*P* < 0.05 and ^∗∗^*P* < 0.01.

EM23 (**Figure 1A**) was isolated and purified from EM by our group. The chemical structure of EM23 was identified by ^1^H-NMR and ^13^C-NMR spectra data as described in our previous study (Liang et al., 2012). A stock solution of EM23 was dissolved in DMSO at concentration of 100 mM and diluted to the indicated final concentration in culture medium. DMSO was diluted to 0.1% in medium as a vehicle control.

The reagents DAPI, DCFH-DA, PI, JC-1, and NAC were purchased from Sigma Chemical Co. (St. Louis, MO, USA). ERK inhibitor FR180204 was purchased from Merck Millipore (Bellerica, MA, USA). A Pierce^TM^ BCA Protein Assay Kit was obtained from Thermo Fisher Scientific (Rockford, IL, USA). TNF-α was obtained from Sino Biological Inc. (Beijing, China). A CCK-8, a TUNEL Apoptosis Detection Kit, dithiothreitol (DTT), a Nuclear and Cytoplasmic Extraction Kit, RIPA buffer and RNase were purchased from Beyotime (Shanghai, China). Phosphatase inhibitor cocktail tablets and protease inhibitor cocktail tablets were supplied by Roche (Mannheim, Germany). All other chemicals and solvents were of reagent or HPLC grade.

Primary antibodies against TrxR, Trx, ASK1, p-ASK1 (Thr845), and Lamin B1 were purchased from Santa Cruz Biotechnology (Dallas, TX, USA). GAPDH, β-actin, caspase 3, caspase 9, cleaved-caspase 3, cleaved-caspase 9, cleaved PARP, p-p38 (Thr180/Tyr182), p-ERK1/2 (Thr202/Tyr204), p-JNK (Thr183/Tyr185), p38, ERK1/2, JNK, p-NF-κB p-p65 (Ser536), NF-κB p65, IκBα, anti-mouse, and anti-rabbit horseradish peroxidase-conjugated secondary antibodies were purchased from Cell Signaling Technology (CST, Beverly, MA, USA). Alexa Fluor^®^ 568 phalloidin and Alexa Fluor^®^ 488 anti-rabbit fluorescent secondary antibodies were purchased from Life Technologies (Grand Island, NY, USA).

### Cell Proliferation Analysis

A CCK-8 assay was used to detect EM23-mediated inhibition of cellular proliferation. Cells in suspension were plated in 96-well plates at a density of 1 × 10^4^ cells/well. Following this, the cells were treated with either vehicle (0.1% DMSO) or EM23. The highest concentration of EM23 used was 100 μM; additional concentrations were tested following two-fold serial dilutions. The cells were treated for 48 h and then 10 μL CCK-8 solution was added to each well, and the plate was incubated for an additional 4 h. The absorbance of the plate was measured at 450 nm using a microplate reader (Bio-Rad; Hercules, CA, USA), and the IC50 values for the different treatment conditions were calculated using Origin 8 software (OriginLab, Northampton, MA, USA).

### Cell Cycle Analysis

K562 (2 × 10^5^ cells/mL) and HL-60 (3 × 10^5^ cells/mL) cells were seeded into 6-well plates and then treated with vehicle or EM23 for 48 h. Following this, the cells were collected, washed twice with PBS, and fixed in cold 70% ethanol (–20°C) overnight. The ethanol was then carefully removed by centrifugation to avoid cell loss, and the cells were resuspended in 1 mL staining reagent (100 mg/mL RNase and 50 mg/mL PI) and kept in darkness for 35 min at room temperature. Cell cycle analysis was accomplished using flow cytometry (BD FACSCalibur, Franklin Lakes, CA, USA) with a wave length of 605 nm.

### Apoptosis Detection via Annexin-V-FITC/PI Assay

K562 (2 × 10^5^ cells/mL) and HL-60 (3 × 10^5^ cells/mL) cells were plated in 6-well plates during the log phase of growth and then treated with either vehicle or the indicated concentrations of EM23 for 48 h. The treated cells were harvested by centrifugation at room temperature and washed twice with ice-cold PBS. Following this, the cells were stained with Annexin-V-FITC/PI (KeyGEN; Nanjing, China) according to the manufacturer’s instructions and analyzed via flow cytometry.

### Western Blotting Analysis

K562 and HL-60 cells were harvested and washed with PBS after treatment with EM23 according to the specific experimental design. Following this, the cells were lysed in RIPA buffer containing 0.5 M DTT, 0.1 M PMSF, 20× protease inhibitor cocktail, and 20× phosphatase inhibitor cocktail for 30 min on ice, and then centrifuged at 12000 *g* at 4°C for 15 min. The resultant supernatants, which contained total cellular protein, were collected, and nuclear proteins were extracted using a nuclear and cytoplasmic extraction kit. The protein concentration was measured using a BCA protein assay kit. Equal amounts of protein (30 μg) were separated via 10–15% gradient SDS-PAGE and transferred to PVDF membranes (Millipore, USA). The membranes were blocked with 5% BSA at room temperature for 1 h, incubated with primary antibodies for at least 16 h at 4°C, and then washed and incubated with HRP-conjugated secondary antibodies at room temperature for 1 h. Protein bands were visualized using enhanced chemiluminescence detection reagents (Bio-Rad, USA). The resulting images were scanned using a scanner (Epson V330 Photo, Japan).

### TUNEL and DAPI Co-staining

K562 and HL-60 cells were treated with either vehicle or EM23 for 48 h, collected into Eppendorf tubes, and washed twice with PBS. Following 4% paraformaldehyde fixation, the cells were permeabilized with 0.2% Triton X-100 in PBS. Next, the cells were stained with TUNEL reaction mixture and DAPI, after which they were washed again with ice-cold PBS. The stained cells were resuspended to a specific cell density and examined under a confocal laser scanning microscope (Zeiss LSM 510, Germany) for evidence of nuclear fragmentation.

### Immunofluorescent Cell Staining

K562 (2 × 10^5^ cells/mL) and HL-60 (3 × 10^5^ cells/mL) cells were seeded in 6-well plates and then co-treated with vehicle or EM23 with or without TNF-α (1 ng/mL) for 1 h. Following this, the cells were collected, and subsequent operations were performed in Eppendorf tubes. First, the cells were washed with ice-cold PBS. Following this, the cells were fixed in 4% paraformaldehyde for 15 min at room temperature and permeabilized with 0.2% Triton X-100 in PBS. After being rinsed with PBS, the cells were blocked with 5% BSA and incubated with an anti-p65 primary antibody and an anti-rabbit fluorescent secondary antibody. Following this, the cells were counterstained with phalloidin and DAPI, washed again with ice-cold PBS and resuspended to a specific cell density. To visualize the nuclear translocation of p65, the above-treated cells were placed into a confocal 35-mm clear cover glass-bottom petri-dish and then viewed and photographed using a confocal laser scanning microscope. The collected images were processed using the manufacturer’s software.

### Evaluation of MMP

Mitochondrial membrane potential was measured using JC-1 dye according to the manufacturer’s instructions. Cells treated with the indicated experimental conditions were harvested and then incubated with JC-1 dye (10 μg/mL) in the dark for 20 min at 37°C. Following this, the cells were washed with ice-cold PBS and analyzed by flow cytometry.

### Determination of Cellular ROS Generation

Intracellular ROS levels were measured using a DCFH-DA assay after EM23 treatment. Briefly, after treatment with EM23 for1–4 h, K562, and HL-60 cells were incubated with 10 μM DCF-DA at 37°C for 30 min and then washed twice and resuspended in PBS. Fluorescence intensity was measured by flow cytometry, and the percentages of fluorescence-positive cells were proportional to the ROS levels within the cell cytosol.

### Statistical Analysis

All data are expressed as the mean ± standard deviation (SD) of three independent experiments. Statistical significance was assessed using Student’s *t*-test (for comparisons of two treatment groups) or one-way ANOVA (for comparisons of three or more groups). *P*-values < 0.05 were considered statistically significant.

## Results

### EM23 Inhibits the Proliferation of K562 and HL-60 Leukemia Cells

The chemical structure of EM23 is shown in **Figure 1A**. A CCK-8 assay was used to evaluate the ability of EM23 to inhibit leukemia cell proliferation. **Figure 1B** shows that EM23 suppressed the proliferation of K562 and HL-60 cells in a dose-dependent manner. After 48 h of treatment with EM23, the IC_50_ values for K562 and HL-60 cells were 10.8 and 1.9 μM, respectively. When the treatment time was extended to 72 h, EM23 showed more significant proliferation inhibitory activities, with IC_50_ values of 6.3 and 1.4 μM for K562 and HL-60 cells, respectively.

We also examined the effects of EM23 on the proliferation of normal mammalian HL-7702 and NIH/3T3 cells. As shown in **Figure 1C**, EM23 exhibited lower proliferation inhibitory activities in HL-7702 and NIH/3T3 cells as compared to those in K562 and HL-60 leukemia cells. The IC_50_ values of EM23 for HL-7702 and NIH/3T3 cells were 40.4 and 25.6 μM, respectively, which were approximately 4-fold and 2-fold higher than that of for K562 cells, and 21-fold and 13-fold higher than that of for HL-60 cells, respectively. In addition, the rate of cell viability was above 85% when HL-7702 and NIH/3T3 cells were treated with EM23 at 8 μM for 48 h.

### EM23 Delays Cell Cycle Progression in K562 and HL-60 Cells

To elucidate whether EM23-mediated decreases in K562 and HL-60 cell proliferation were associated with changes in cell cycle progression, the cell cycle phase distributions of EM23-treated cells were analyzed by flow cytometry. As shown in **Figure 1C**, treatment with increasing concentrations of EM23 for 48 h decreased the G_0_/G_1_ phase DNA content from 42.6 to 35.6%, whereas the G_2_/M DNA content increased from 5.1 to 12.5% in K562 cells, which dose-dependently accumulated the cells in G2/M phase. In HL-60 cells, treatment with 4 μM EM23 increased the number of cells in S phase by 29.5%, while the number of cells in G_0/_G_1_ phase decreased by 23.1% compared with the control (**Figure 1D**).

### EM23 Induces Apoptosis and Caspase Activation in K562 and HL-60 Cells

**FIGURE 2:**
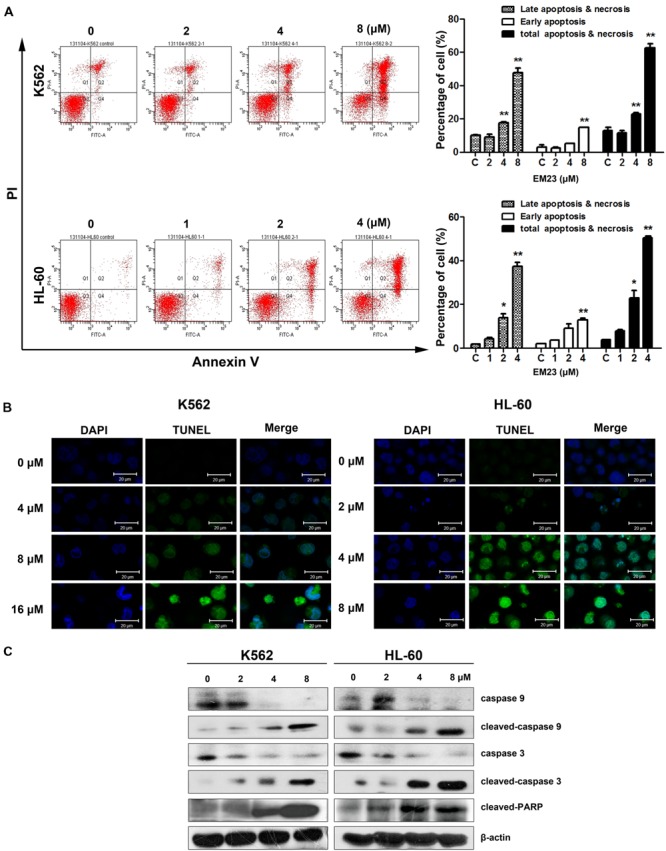
**EM23 induced cell apoptosis. (A)** Effects of EM23 on the induction of apoptosis as measured by flow cytometry. K562 and HL-60 cells were treated with EM23 for 48 h, and the apoptotic cell rate was analyzed after Annexin-V-FITC/PI staining. The data are presented as the mean ± SD of three independent experiments. ^∗^*P* < 0.05 and ^∗∗^*P* < 0.01. **(B)** Representative images of DNA fragmentation and nuclear condensation in K562 and HL-60 cells exposed to EM23. Nuclei were detected by TUNEL and DAPI staining. All images shown are representative of three independent experiments. **(C)** Western blotting analysis of caspase 9, cleaved caspase 9, caspase 3, cleaved caspase 3, and cleaved PARP (89 kDa) expression; β-actin served as an internal control. The data shown are representative of three independent experiments.

To further investigate the role of EM23 in the induction of apoptotic cell death, K562 and HL-60 cells were treated with the indicated concentrations of EM23 for 48 h, and cell apoptosis was assessed via Annexin-V-FITC/PI FACS analysis. Notably, both cell lines showed significant dose-dependent increases in apoptosis ratios following treatment with EM23 (**Figure 2A**). At an EM23 concentration of 8 μM, the apoptosis ratios were 62.6% for K562 cells. In HL-60 cells, treatment with 4 μM EM23 increased the percentage of apoptotic cells by 46.5% compared with the control (**Figure 2A**). Furthermore, TUNEL-DAPI co-staining revealed that the EM23-treated cells exhibited enhanced apoptotic features, such as DNA fragmentation and nuclear condensation (**Figure 2B**).

We next analyzed caspase activation and PARP cleavage in EM23-treated cells. As shown in **Figure 2C**, the levels of initiator caspases 9 and 3 significantly decreased as the concentration of EM23 increased. Additionally, the levels of cleaved caspase 9 and 3 dose-dependently increased with EM23 treatment. PARP, a DNA-repair enzyme, serves as a substrate for caspase 3 (Mullen, 2004). We next examined PARP degradation following EM23 treatment. As shown in **Figure 2C**, EM23 treatment led to specific proteolytic cleavage of PARP, as indicated by the presence of 116 to 89 kDa PARP fragments in K562 and HL-60 cells. These results suggest that caspase activation and PARP cleavage may underlie EM23-mediated apoptosis in K562 and HL-60 cells.

### EM23 Induces a Loss in MMP

**FIGURE 3:**
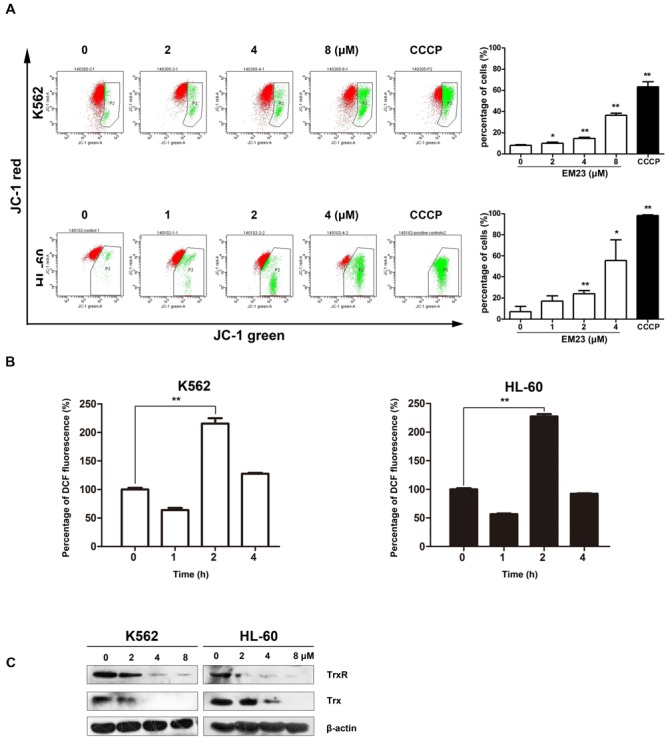
**EM23 interfered with cellular redox homeostasis. (A)** Flow cytometry analysis of MMP based on JC-1 staining. Cells were treated with EM23 for 48 h and stained with JC-1. The cells showing a loss of MMP were gated. Cells were exposed to the MMP disrupter carbonyl cyanide 3-chlorophenylhydrazone (CCCP, 10 μM) for 20 min as a positive control. All data are presented as the mean ± SD of three independent experiments. ^∗^*P* < 0.05 and ^∗∗^*P* < 0.01. **(B)** EM23 induced ROS production in K562 and HL-60 cells. Cells were treated with 2 μM EM23 for 1–4 h, stained with DCFH-DA for 20 min, and analyzed for fluorescence by flow cytometry. All data are presented as the mean ± SD of three independent experiments. ^∗^*P* < 0.05 and ^∗∗^*P* < 0.01. **(C)** Effects of EM23 on the expression levels of Trx and TrxR. K562 and HL-60 cells were treated with EM23 for 48 h and then subjected to western blotting. β-actin was used as an internal control. The data shown are representative of three independent experiments.

The loss of MMP (Δψ_m_) is a vital cellular event during the process of intrinsic apoptosis. Therefore, we measured variations in Δψ_m_ using a JC-1 MMP assay to monitor mitochondrial status in EM23-treated K562 and HL-60 cells. As shown in **Figure 3A**, EM23 treatment clearly disrupted Δψ_m_ in both cell lines in a dose-dependent manner. The proportions of green-fluorescent cells within K562 and HL-60 cell populations increased by 36.2 and 55.5%, respectively, following treatment with 8 and 4 μM EM23, demonstrating that EM23 led to MMP disruption in both cell lines.

### EM23 Increases Cellular ROS Levels and Suppresses Trx System

To investigate whether EM23-mediated apoptosis was related to the presence of ROS, we examined intracellular ROS production in EM23-treated K562 and HL-60 cells using the fluorescent probe DCFH-DA. As shown in **Figure 3B**, compared to the control, intracellular ROS levels increased by over twofold in both cell lines following exposure to EM23 for 2 h, indicating that ROS accumulation occurred in both K562 and HL-60 cells in response to treatment with EM23. Interestingly, continuous incubation with EM23 for 4 h led to decreased ROS levels in both cell lines relative to those measured after 2 h of treatment.

**FIGURE 4:**
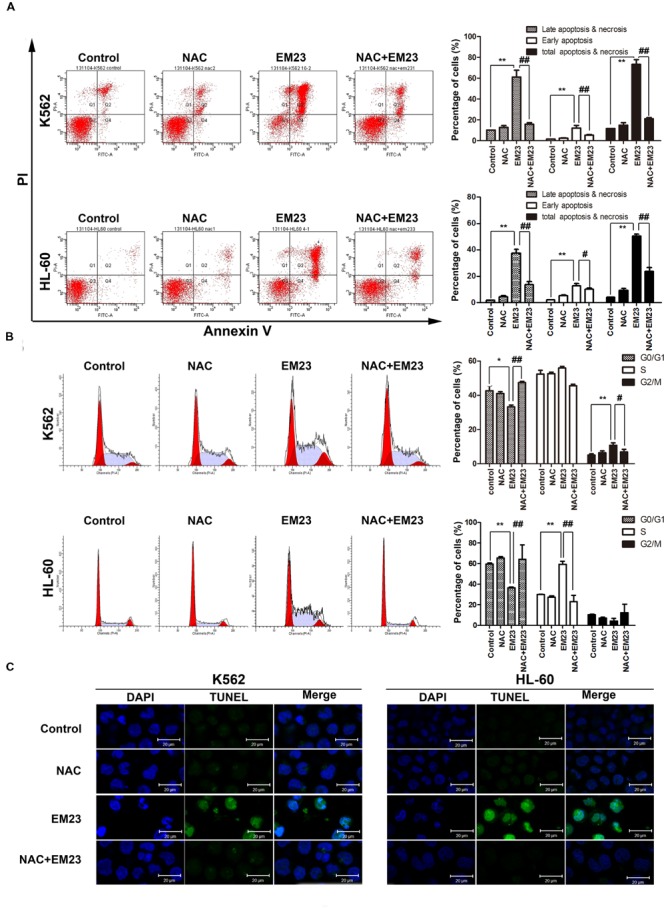
**NAC reversed EM23-mediated apoptosis and cells accumulation. (A)** The induction of apoptosis in K562 and HL-60 cells following treatment with EM23 was rescued following treatment with NAC. K562 and HL-60 cells were pretreated with 5 mM NAC for 1 h, followed by treatment with 8 and 4 μM EM23 for 48 h, respectively. Next, the treated cells were stained with Annexin-V-FITC/PI and analyzed by flow cytometry. All data are presented as the mean ± SD of three independent experiments. Bars with different characters indicate significant differences. ^∗^*P* < 0.05 and ^∗∗^*P* < 0.01 compared to control; ^#^*P* < 0.05 and ^##^*P* < 0.01 compared to NAC+EM23. **(B)** NAC inhibited EM23-mediated cell cycle redistribution. K562 and HL-60 cells were pretreated with 5 mM NAC for 1 h, followed by treatment with 8 and 4 μM EM23 for 48 h, respectively. The cell cycle phase distributions of the EM23-treated cells were analyzed by flow cytometry. All data are presented as the mean ± SD of three independent experiments. Bars with different characters indicate significant differences. ^∗^*P* < 0.05 and ^∗∗^*P* < 0.01 compared to control; ^#^*P* < 0.05 and ^##^*P* < 0.01 compared to NAC+EM23. **(C)** NAC reduced the morphological changes caused by EM23. K562 and HL-60 cells were treated with 16 and 8 μM, respectively, and then subjected to TUNEL-DAPI co-staining and examined under a confocal laser scanning microscope. All images shown are representative of three independent experiments that produced similar results.

To further investigate the role of ROS generation in EM23-mediated apoptosis, both cell lines were pretreated with the ROS scavenger NAC. Flow cytometry analysis demonstrated that EM23-mediated apoptosis and accumulation of cells in G_2_/M or S phase were almost entirely rescued by NAC (**Figures 4A,B**). As shown in **Figure 4C**, pretreatment with NAC also significantly inhibited EM23-mediated DNA fragmentation and nuclear condensation based on TUNEL-DAPI co-staining. These results suggest that ROS accumulation was required for apoptotic cell death in EM23-treated K562 and HL-60 cells.

The Trx system, one of the major intracellular antioxidant systems, is integral for maintaining a balanced intracellular redox state (Collet and Messens, 2010). The effects of EM23 on Trx and TrxR expression were examined in K562 and HL-60 cells. As shown in **Figure 3C**, the expression levels of both Trx and TrxR were dose-dependently down-regulated by EM23 in K562 and HL-60 cells. These results suggest that EM23 may increase cellular ROS levels by inhibiting the Trx system.

### ASK1- and MAPK-Mediated Signaling Pathways in EM23-Treated Cells

**FIGURE 5:**
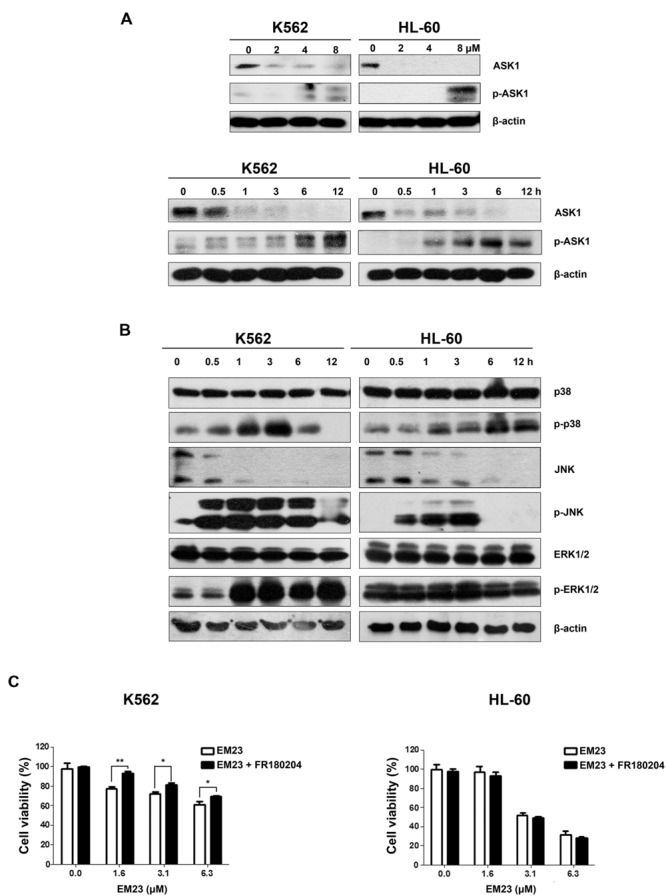
**Effects of EM23 on ASK1 and p38/JNK/ERK MAPK activation. (A)** K562 and HL-60 cells were treated with 0, 2, 4, and 8 μM EM23 for 48 h or treated with 8 μM EM23 for 0, 0.5, 1, 3, 6, and 12 h. The expression levels of ASK1 and p-ASK1 were analyzed by western blotting analysis. β-actin was used as an internal control. **(B)** K562 and HL-60 cells were exposed to 8 μM EM23 for 0, 0.5, 1, 3, 6, and 12 h and then the phosphorylation levels of p38, JNK, and ERK1/2 were detected by western blotting. All protein levels were normalized to the level of β-actin. The data shown are representative of three independent experiments. **(C)** Effects of ERK inhibitor FR180204 on EM23-mediated proliferation inhibitory activities. K562 and HL-60 cells were pre-treated with 5 and 2.5 μM FR180204, respectively. Then the cells were incubated with various concentrations of EM23 for 48 h. Cell viability was measured using a CCK-8 assay. The data shown are the mean ± SD of at least three independent experiments. ^∗^*P* < 0.05 and ^∗∗^*P* < 0.01.

ASK1 is activated by various types of stress, including the accumulation of ROS. The reduced form of Trx is an endogenous inhibitor of ASK1. When Trx is oxidized, it dissociates from ASK1, which is then activated by the autophosphorylation of Thr845 in its kinase domain, leading to the downstream activation of the JNK and p38 MAPK pathways (Lu and Holmgren, 2012). As shown in **Figure 5A**, EM23 activated endogenous ASK1 through the phosphorylation of its Thr845 residue in a time-dependent and dose-dependent manner in K562 and HL-60 cells. It is interesting that ASK1 expression dose-dependently and time-dependently decreased following treatment with EM23 (**Figure 5A**). Next, we investigated how treatment with EM23 affected the phosphorylation statuses of three different MAPKs (p38, JNK, and ERK1/2) in K562 and HL-60 cells by western blotting analysis. As shown in **Figure 5B** p38 and JNK were time-dependently activated in both cell lines following treatment with 8 μM EM23. The maximum activation of JNK by EM23 occurred within 3 h of treatment, followed by a progressive decline after 6 h (**Figure 5B**). A similar progression of p38 activation was also observed in EM23-treated K562 cells, while continuous activation of p38 was observed in EM23-treated HL-60 cells (**Figure 5B**). K562 and HL-60 cells showed different ERK1/2 phosphorylation profiles following EM23 treatment (**Figure 5B**). Treatment of K562 cells with 8 μM EM23 markedly increased ERK1/2 phosphorylation within 1 h, followed by sustained activation of ERK1/2 within 12 h. Unlike in K562 cells, no obvious activation of ERK1/2 was observed in HL-60 cells treated with EM23 for 12 h (**Figure 5B**). The ERK inhibitor FR180204 was further used to clarify the role of ERK in EM23-mediated apoptosis. As shown in **Figure 5C**, co-incubation of EM23 and FR180204 significantly increased the viability of K562 cells as compared to the cells only treated with EM23. While there was no obvious change in cellular viability when HL-60 cells were co-treated with EM23 and FR180204. These results indicated that MAPK activation might be involved in EM23-mediated apoptosis.

**FIGURE 6:**
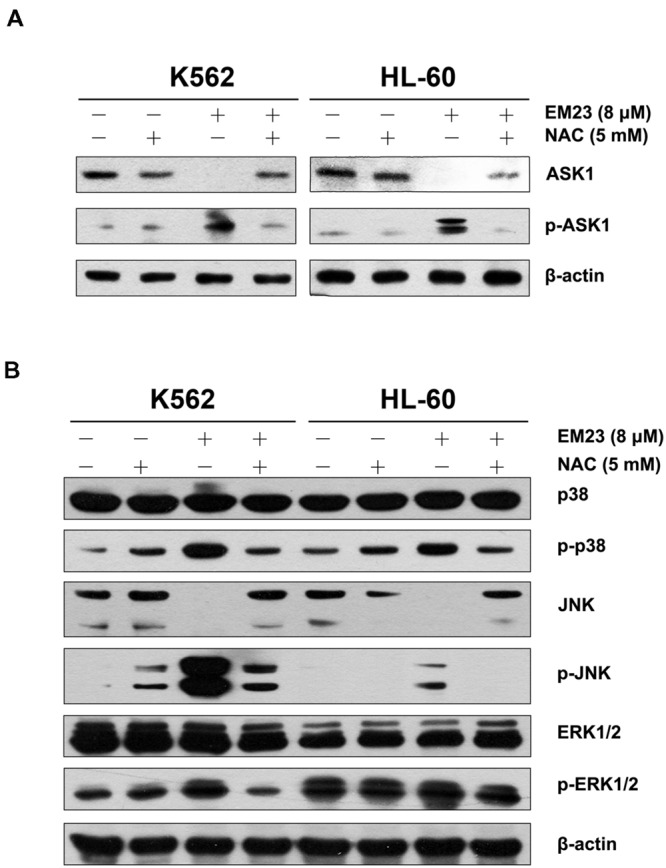
**NAC reversed EM23-mediated increases in ASK1 and MAPK phosphorylation. (A)** K562 and HL-60 cells were treated with vehicle, 5 mM NAC, or 8 μM EM23 for 12 h. Cells were also pretreated with 5 mM NAC for 1 h, followed by treatment with 8 μM EM23 for 12 h. Protein levels of ASK1 and p-ASK1 were analyzed by western blotting. **(B)** K562 and HL-60 cells were treated with vehicle, 5 mM NAC, and 8 μM EM23 for 3 h. Cells were also pretreated with 5 mM NAC for 1 h, followed by treatment with 8 μM EM23 for 3 h. Total protein extracts from treated cells were submitted to western blotting for detection of p38, p-p38, JNK, p-JNK, ERK1/2, and p-ERK1/2. β-actin was used as an internal control. All data shown are representative of three independent experiments.

To further determine whether ROS production can trigger ASK1 and MAPK activation following EM23 treatment, cells were pretreated with 5 mM NAC for 1 h and then treated with EM23 and subjected to western blotting analysis. As shown in **Figures 6A,B**, NAC blocked EM23-mediated activation of ASK1, p38 and JNK in K562 and HL-60 cells. Similarly, EM23-mediated promotion of ERK1/2 phosphorylation in K562 cells was significantly suppressed following NAC pretreatment, while ERK1/2 phosphorylation in HL-60 cells was unaltered, which was consistent with previous results (**Figure 6B**). These results further support the important role of ROS in EM23-mediated activation of ASK1/MAPK signaling pathways.

### EM23 Suppresses TNF-α-Mediated NF-κB Activation

**FIGURE 7:**
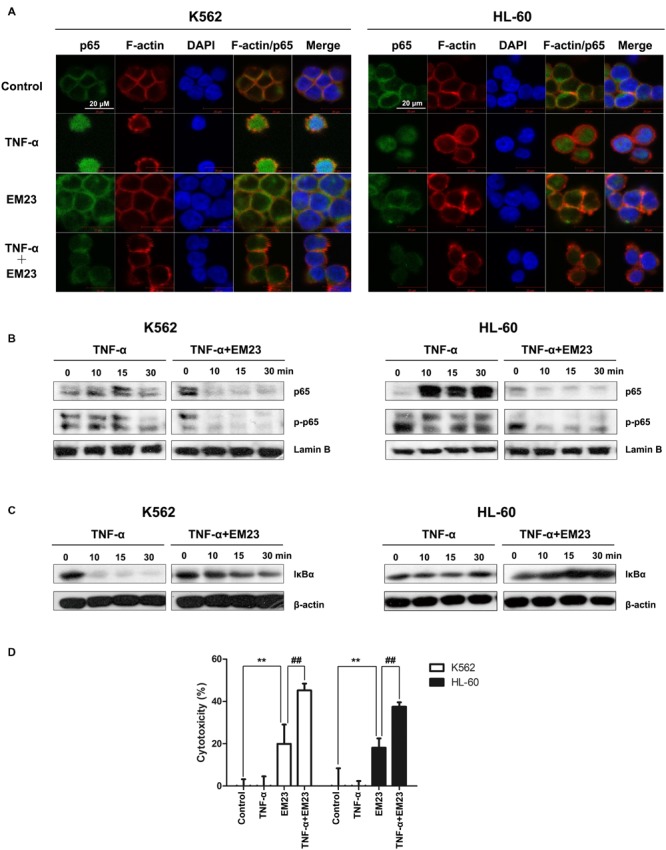
**Inhibitory effects of EM23 on TNF-α-mediated NF-κB activation. (A)** Immunofluorescence analysis of p65 localization. K562 and HL-60 cells were treated with vehicle or EM23 for 1 h and then stimulated with 1 nM TNF-α for 30 min. The cells were stained with DAPI (blue), FITC-labeled anti-p65 antibody (green) and phalloidin (for F-actin labeling, red). All data shown are representative of three independent experiments. **(B)** Effects of EM23 on TNF-α-mediated translocalization and phosphorylation of p65. K562 and HL-60 cells were co-incubated with 8 μM EM23 and 1 nM TNF-α for the indicated times. Nuclear extracts were prepared and submitted to western blotting for the detection of p65 and p-p65. Lamin B1 was used as a loading control. All data shown are representative of three independent experiments. **(C)** Effect of EM23 on TNF-α-mediated degradation of IκBα. K562 and HL-60 cells were co-incubated with 8 μM EM23 and 1 nM TNF-α for the indicated times. Cytoplasmic extracts were prepared and submitted to western blotting for the detection of IκBα. β-actin was used as a loading control. All data shown are representative of three independent experiments. **(D)** EM23 enhanced TNF-α-mediated cytotoxicity. K562 and HL-60 cells were, respectively, incubated with 4 and 2 μM EM23 for 1 h, followed by treatment with 1 nM TNF-α for 24 h. The cytotoxicity of the combined treatment of EM23 and TNF-α was detected by CCK-8 assay. All data are presented as the mean ± SD of three independent experiments. Bars with different characters indicate significant differences. ^∗^*P* < 0.05 and ^∗∗^*P* < 0.01 compared to control; ^#^*P* < 0.05 and ^##^*P* < 0.01 compared to TNF-α+EM23.

Reactive oxygen species interacts with NF-κB signaling pathways in many ways (Morgan and Liu, 2011). Therefore, we investigated the effects of EM23 on TNF-α-mediated NF-κB activation via immunofluorescent staining and western blotting analysis. As shown in **Figure 7A**, p65 was primarily distributed throughout the cytoplasm of the control cells. The addition of TNF-α caused p65 to undergo nuclear translocation, which was significantly blocked following EM23 treatment in both K562 and HL-60 cells (**Figures 7A,B**). Moreover, EM23 treatment significantly suppressed TNF-α-mediated phosphorylation of p65 (**Figure 7B**). In the cytoplasm, TNF-α stimulation decreased levels of cytoplasmic IκBα, while EM23 treatment significantly reversed TNF-α-mediated IκBα degradation, although not completely (**Figure 7C**). These results indicate that EM23 suppresses TNF-α-mediated NF-κB activation.

Next, we examined the effects of combined treatment with TNF-α and EM23 on the proliferation of K562 and HL-60 cells using a CCK-8 assay. As shown in **Figure 7D**, treating cells with a combination of TNF-α and EM23 resulted in higher cytotoxicity, with inhibition rates ranging from 20.0 to 45.2% and from 18.1 to 37.5% in K562 and HL-60 cells, respectively. These results indicated that EM23 is a potent NF-κB inhibitor and that this effect is enhanced by TNF-α-mediated cytotoxicity.

## Discussion

Natural products, in addition to their traditional uses, have served as important sources of bioactive compounds that have played significant roles in medical discovery and development processes (Newman and Cragg, 2012; Wang et al., 2015). The genus *Elephantopus*, which belongs to the Asteraceae family, has recently attracted a great deal of interest due to its diverse pharmacological activities. Several species of *Elephantopus* contained sesquiterpene lactones have been reported to exert cytotoxicity and possess potential anticancer activities. Deoxyelephantopin and isodeoxyelephantopin, two germacranolidese squiterpene lactones, are components of extracts of *Elephantopus scaber* L.. Isodeoxyelephantopin has been reported to suppress NF-κB, potentiate apoptosis, inhibit invasion and abolish osteoclastogenesis in leukemia KBM-5 cells (Ichikawa et al., 2006). Deoxyelephantopin was found to inhibit TS/A tumor growth and lung metastasis and double survival time in mice bearing mammary tumors (Huang et al., 2010). Some of other sesquiterpene lactones from the other Asteraceae plants, such as thapsigargin, artemisinin and parthenolide, have also been reported to have anticancer activities *in vivo*. Thapsigargin derivation G-202 is used for treating breast, kidney and prostate cancer in phase I study (Ghantous et al., 2010). Artemisnin and its derivations, artesunate and artemether, are being tested in phase I/II clinical trials for the treatment of breast, colorectal, and non-small cell lung cancers (Berger et al., 2005; Zhang et al., 2008). A parthenolide analog dimethylamino-parthenolide is now in clinical phase I against AML, ALL, and other blood cancers (Guzman et al., 2007; Ghantous et al., 2010). These findings provide clues for potential therapeutic application of EM23 as anti-leukemia agents in clinic. It is worthy further investigating the effect of EM23 on AML and CML *in vivo* in next study.

Apoptosis, a programmed form of cell death, involves the degradation of cellular constituents by a group of cysteine proteases called caspases. Caspase activation occurs through two main molecular pathways, known as the extrinsic and the intrinsic apoptotic pathways (Burgess, 2013). The intrinsic apoptotic pathway is characterized by mitochondrial permeabilization, causing the release of pro-apoptotic proteins into the cytosol (Matsuzawa and Ichijo, 2008). Indeed, we found that EM23 significantly decreased Δψ_m_ in K562 and HL-60 cells (**Figure 3A**) and up-regulated caspase signaling cascades (caspases 9 and 3), which resulted in the activation of the downstream cellular death substrate PARP (**Figure 2C**). These findings indicate that EM23 may induce apoptosis through the intrinsic apoptotic pathway.

Excessive intracellular production of ROS is known to induce apoptosis (Sabharwal and Schumacker, 2014). The mitochondrial respiratory chain (electron transport complexes) is a major source of intracellular ROS generation (Sabharwal and Schumacker, 2014). In the current study, EM23 induced obvious mitochondrial dysfunction and markedly increased intracellular ROS levels in K562 and HL-60 cells. Cellular ROS generation played a vital role in EM23-mediated apoptosis and accumulation of cells in G2/M or S phase in both cell lines, and co-treatment with EM23 and the reducing agent NAC, which quenches ROS, almost completely reversed these effects (**Figure 4**). These results indicate that EM23-mediated apoptosis in K562 and HL-60 cells might be governed by ROS-mediated mechanisms.

The Trx system, a ubiquitous oxidoreductase system that is overexpressed in various tumor types, has important roles in reducing enzymes and maintaining intracellular protein thiol redox balance (Fink et al., 2015). The mammalian TrxR protein contains a highly reactive active selenocysteine residue that is susceptible to electrophilic compounds and has been identified as a novel chemotherapeutic target for anticancer drug development (Liu et al., 2014). Recent studies have revealed that redox-regulating mechanisms, such as the Trx system, represent important targets for the treatment of malignancies (Trachootham et al., 2009). Disruption of the Trx system by EM23 may interfere with intracellular redox balance and lead to the accumulation of ROS, which subsequently initiates apoptosis in K562 and HL-60 cells.

Trx target proteins are involved in various specific pathways associated with the regulation of apoptosis. ASK1, a MAPKKK family member, is tightly controlled by Trx and acts as a major contributor in regulating ROS-mediated apoptosis through the activation of the JNK and p38 signaling pathways (Seong et al., 2015). Our results demonstrated that EM23 treatment increased the phosphorylation of ASK1 at Thr845 (**Figure 5A**), which further phosphorylated the downstream MAPKs JNK and p38 (**Figure 5B**), suggesting that EM23 may activate the ASK1-JNK/p38 signaling axis in a ROS-dependent manner to exert its pro-apoptotic effects. In addition, following co-treatment with EM23 and NAC, the phosphorylation levels of ASK1, p38 and JNK were notably lower than those following EM23 treatment alone (**Figure 6**). These results are consistent with the full reversion of EM23-mediated apoptosis that was found following pretreatment with NAC. Our results indicated that EM23-mediated ROS accumulation was an important signaling intermediate in ASK1-dependent apoptosis.

It has been well established that increases in ROS levels are effective inducers of MAPK-mediated apoptosis (Finkel and Holbrook, 2000). As shown in **Figure 6B**, downstream MAPK (p38, JNK and ERK1/2) pathways became activated following EM23 treatment in K562 cells. In HL-60 cells, treatment with EM23 also led to significant activation of p38 and JNK; however, there was no obvious increase in ERK1/2 phosphorylation during the treatment (**Figure 6B**). Our results are in agreement with other studies reporting that compounds that cause ROS generation do not necessarily lead to increases in ERK1/2 phosphorylation in treated HL-60 cells (Uen et al., 2007; Wang et al., 2014). It is well documented that the activation of ERK1/2 generally promotes cell survival. However, depending on cell type and stimulus, ERK1/2 also can mediate pro-apoptotic functions (Cagnol and Chambard, 2010; Li et al., 2013). Our results indicated that ERK1/2 activation in K562 cells may contribute to EM23-mediated apoptosis (**Figure 5C**).

NF-κB, one of major chemoresistance-related anti-apoptotic factors (Yip et al., 2011), is frequently up-regulated in human chronic and AML (Hsieh and Van Etten, 2014). Trx plays dual and opposing roles in the regulation of NF-κB: in the cytoplasm, Trx blocks IκBα degradation, thereby inhibiting NF-κB activation (Takeuchi et al., 2000), whereas in the nucleus Trx enhances NF-κB transcriptional activity by enhancing its ability to bind DNA (Hirota et al., 1999; Ueno et al., 2007). In the current study, it is possible that EM23 inhibited the NF-κB pathway by inhibiting the Trx system. Moreover, small molecules that mimic EM23 by simultaneously activating ROS-MAPK pro-apoptotic pathways and blocking anti-apoptotic NF-κB pathways may improve the outcomes of myeloid leukemia chemotherapy.

**FIGURE 8:**
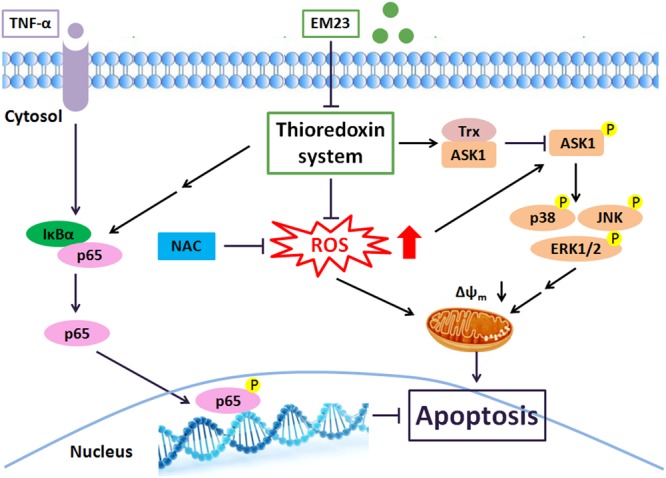
**EM23 mediation of intracellular signaling leading to apoptosis in human myeloid leukemia cells.** Key: black arrows, activation; black blocks, inhibition; double arrows, multiple signal transduction steps.

In summary, we showed the ability of EM23 to inhibit the proliferation of human myeloid leukemia K562 and HL-60 cells by inducing apoptosis via mitochondrial pathways. Suppression of the Trx system following treatment with EM23 could result in ROS accumulation and subsequently activate a number of Trx-dependent pathways, including the ASK1, p38, JNK and ERK MAPK pathways, which may contribute to EM23-mediated apoptosis (**Figure 8**). Furthermore, EM23 also effectively blocks TNF-α/NF-κB axis signaling, implying that EM23 has a potential role in preventing NF-κB-mediated anti-apoptotic pathways. This report represents the first comprehensive analysis of the cellular signaling events that are affected by treatment with EM23, a compound found in EM, in human myeloid leukemia cells. The findings contained here in detail a possible anticarcinogenic molecular mechanism for EM23.

## Author Contributions

Participated in research design: HL, ZL, FS, GZ. Conducted experiments: HL, ML, CX, FS, SW, WC. Contributed new reagents or analytic tools: YL, GZ. GW, ML. Performed data analysis: HL, ZL, FS, CX, YL, GZ. Wrote or contributed to the writing of the manuscript: HL, ML, YL, GZ, ZL.

## Conflict of Interest Statement

The authors declare that the research was conducted in the absence of any commercial or financial relationships that could be construed as a potential conflict of interest.

## Abbreviations

ASK1: apoptosis regulating-signal kinase 1
CCK-8: Cell Counting Kit-8
DAPI: 4′,6-diamidino-2-phenylindole
DCFH-DA: 2’,7’-dichlorofluorescein diacetate
DMSO: dimethyl sulfoxide
EM: *Elephantopus mollis*
JC-1: 5,5′,6,6′-tetrachloro-1,1′,3,3′-tetraethyl-imidacarbocyanine iodide
MAPK: mitogen-activated protein kinases
MMP: mitochondrial membrane potential
MTT: 3-(4,5-dimethylthiazol-2-yl)-2,5-diphenyltetrazolium bromide
NAC: *N*-acetyl-L-cysteine
NF-κB: nuclear factor-κB
PI: propidium iodide
ROS: reactive oxygen species
TKIs: tyrosine kinase inhibitors
TNF-α: tumor necrosis factor alpha
Trx: thioredoxin
TrxR: thioredoxinreductase
TUNEL: terminal deoxynucleotidyltransferase-mediated dUTP nick-end labeling

## References

[B1] Berger T. G. Dieckmann D. Efferth T. Schultz E. S. Funk J. O. Baur A. (2005). Artesunate in the treatment of metastatic uveal melanoma–first experiences. *Oncol. Rep.* 14 1599–1603. 10.3892/or.14.6.1599 16273263

[B2] Bhamidipati P. K. Kantarjian H. Cortes J. Cornelison A. M. Jabbour E. (2013). Management of imatinib-resistant patients with chronic myeloid leukemia. *Ther. Adv. Hematol.* 4 103–117. 10.1177/2040620712468289 23610618 PMC3629755

[B3] Burgess D. J. (2013). Apoptosis: refined and lethal. *Nat. Rev. Cancer* 13 79 10.1038/nrc3462 23344537

[B4] Cagnol S. Chambard J. C. (2010). ERK and cell death: mechanisms of ERK-induced cell death–apoptosis, autophagy and senescence. *FEBS J.* 277 2–21. 10.1111/j.1742-4658.2009.07366.x 19843174

[B5] Collet J. F. Messens J. (2010). Structure, function, and mechanism of thioredoxin proteins. *Antioxid. Redox Signal.* 13 1205–1216. 10.1089/ars.2010.3114 20136512

[B6] Degos L. Wang Z. Y. (2001). All trans retinoic acid in acute promyelocytic leukemia. *Oncogene* 20 7140–7145. 10.1038/sj.onc.1204763 11704842

[B7] Dohner H. Estey E. H. Amadori S. Appelbaum F. R. Buchner T. Burnett A. K. (2010). Diagnosis and management of acute myeloid leukemia in adults: recommendations from an international expert panel, on behalf of the European LeukemiaNet. *Blood* 115 453–474. 10.1182/blood-2009-07-235358 19880497

[B8] Fink E. E. Mannava S. Bagati A. Bianchi-Smiraglia A. Nair J. R. Moparthy K. (2015). Mitochondrial thioredoxin reductase regulates major cytotoxicity pathways of proteasome inhibitors in multiple myeloma cells. *Leukemia* 30 104–111. 10.1038/leu.2015.190 26205085 PMC5436048

[B9] Finkel T. Holbrook N. J. (2000). Oxidants, oxidative stress and the biology of ageing. *Nature* 408 239–247. 10.1038/35041687 11089981

[B10] Gachet M. S. Lecaro J. S. Kaiser M. Brun R. Navarrete H. Munoz R. A. (2010). Assessment of anti-protozoal activity of plants traditionally used in Ecuador in the treatment of leishmaniasis. *J. Ethnopharmacol.* 128 184–197. 10.1016/j.jep.2010.01.007 20064594

[B11] Ghantous A. Gali-Muhtasib H. Vuorela H. Saliba N. A. Darwiche N. (2010). What made sesquiterpene lactones reach cancer clinical trials? *Drug Discov. Today* 15 668–678. 10.1016/j.drudis.2010.06.002 20541036

[B12] Goldman J. M. Majhail N. S. Klein J. P. Wang Z. Sobocinski K. A. Arora M. (2010). Relapse and late mortality in 5-year survivors of myeloablative allogeneic hematopoietic cell transplantation for chronic myeloid leukemia in first chronic phase. *J. Clin. Oncol.* 28 1888–1895. 10.1200/JCO.2009.26.7757 20212247 PMC2860369

[B13] Grimwade D. Hills R. K. Moorman A. V. Walker H. Chatters S. Goldstone A. H. (2010). Refinement of cytogenetic classification in acute myeloid leukemia: determination of prognostic significance of rare recurring chromosomal abnormalities among 5876 younger adult patients treated in the United Kingdom Medical Research Council trials. *Blood* 116 354–365. 10.1182/blood-2009-11-254441 20385793

[B14] Guzman M. L. Rossi R. M. Neelakantan S. Li X. Corbett C. A. Hassane D. C. (2007). An orally bioavailable parthenolide analog selectively eradicates acute myelogenous leukemia stem and progenitor cells. *Blood* 110 4427–4435. 10.1182/blood-2007-05-090621 17804695 PMC2234793

[B15] Hanahan D. Weinberg R. A. (2011). Hallmarks of cancer: the next generation. *Cell* 144 646–674. 10.1016/j.cell.2011.02.013 21376230

[B16] Hasegawa K. Furuya R. Mizuno H. Umishio K. Suetsugu M. Sato K. (2010). Inhibitory effect of *Elephantopus mollis* H.B. and K. extract on melanogenesis in B16 murine melanoma cells by downregulating microphthalmia-associated transcription factor expression. *Biosci. Biotechnol. Biochem.* 74 1908–1912. 10.1271/bbb.100318 20834163

[B17] Hirota K. Murata M. Sachi Y. Nakamura H. Takeuchi J. Mori K. (1999). Distinct roles of thioredoxin in the cytoplasm and in the nucleus. A two-step mechanism of redox regulation of transcription factor NF-kappaB. *J. Biol. Chem.* 274 27891–27897. 10.1074/jbc.274.39.27891 10488136

[B18] Hsieh M. Y. Van Etten R. A. (2014). IKK-dependent activation of NF-kappaB contributes to myeloid and lymphoid leukemogenesis by BCR-ABL1. *Blood* 123 2401–2411. 10.1182/blood-2014-01-547943 24464015 PMC3983614

[B19] Huang C. C. Lo C. P. Chiu C. Y. Shyur L. F. (2010). Deoxyelephantopin, a novel multifunctional agent, suppresses mammary tumour growth and lung metastasis and doubles survival time in mice. *Br. J. Pharmacol.* 159 856–871. 10.1111/j.1476-5381.2009.00581.x 20105176 PMC2829211

[B20] Hughes T. Deininger M. Hochhaus A. Branford S. Radich J. Kaeda J. (2006). Monitoring CML patients responding to treatment with tyrosine kinase inhibitors: review and recommendations for harmonizing current methodology for detecting BCR-ABL transcripts and kinase domain mutations and for expressing results. *Blood* 108 28–37. 10.1182/blood-2006-01-0092 16522812 PMC1895821

[B21] Ichikawa H. Nair M. S. Takada Y. Sheeja D. B. Kumar M. A. Oommen O. V. (2006). Isodeoxyelephantopin, a novel sesquiterpene lactone, potentiates apoptosis, inhibits invasion, and abolishes osteoclastogenesis through suppression of nuclear factor-kappaB (nf-kappaB) activation and nf-kappaB-regulated gene expression. *Clin. Cancer Res.* 12 5910–5918. 10.1158/1078-0432.CCR-06-0916 17021000

[B22] Kantarjian H. Sawyers C. Hochhaus A. Guilhot F. Schiffer C. Gambacorti-Passerini C. (2002). Hematologic and cytogenetic responses to imatinib mesylate in chronic myelogenous leukemia. *N. Engl. J. Med.* 346 645–652. 10.1056/NEJMoa011573 11870241

[B23] Li F. Jiang Q. Shi K. J. Luo H. Yang Y. Xu C. M. (2013). RhoA modulates functional and physical interaction between ROCK1 and Erk1/2 in selenite-induced apoptosis of leukaemia cells. *Cell Death Dis.* 4:e708 10.1038/cddis.2013.243 PMC3730416 23828571

[B24] Liang N. Yang X. X. Wang G. C. Wu X. Yang Y. T. Luo H. J. (2012). [Study on the chemical constituents of *Elephantopus mollis*]. *Zhong Yao Cai* 35 1775–1778. 23627086

[B25] Lin C. C. Tsai C. C. Yen M. H. (1995). The evaluation of hepatoprotective effects of Taiwan folk medicine ‘teng-khia-u.’ *J. Ethnopharmacol.* 45 113–123. 10.1016/0378-8741(94)01198-9 7776660

[B26] Liu Y. Duan D. Yao J. Zhang B. Peng S. Ma H. (2014). Dithiaarsanes induce oxidative stress-mediated apoptosis in HL-60 cells by selectively targeting thioredoxin reductase. *J. Med. Chem.* 57 5203–5211. 10.1021/jm500221p 24867309

[B27] Lu J. Holmgren A. (2012). Thioredoxin system in cell death progression. *Antioxid. Redox. Signal.* 17 1738–1747. 10.1089/ars.2012.4650 22530689

[B28] Matsuzawa A. Ichijo H. (2008). Redox control of cell fate by MAP kinase: physiological roles of ASK1-MAP kinase pathway in stress signaling. *Biochim. Biophys. Acta* 1780 1325–1336. 10.1016/j.bbagen.2007.12.011 18206122

[B29] Morgan M. J. Liu Z. G. (2011). Crosstalk of reactive oxygen species and NF-kappaB signaling. *Cell Res.* 21 103–115. 10.1038/cr.2010.178 21187859 PMC3193400

[B30] Mullen P. (2004). PARP cleavage as a means of assessing apoptosis. *Methods Mol. Med.* 88 171–181. 14634228 10.1385/1-59259-406-9:171

[B31] Newman D. J. Cragg G. M. (2012). Natural products as sources of new drugs over the 30 years from 1981 to 2010. *J. Nat. Prod.* 75 311–335. 10.1021/np200906s 22316239 PMC3721181

[B32] Ngueguim F. T. Khan M. P. Donfack J. H. Siddiqui J. A. Tewari D. Nagar G. K. (2012). Evaluation of Cameroonian plants towards experimental bone regeneration. *J. Ethnopharmacol.* 141 331–337. 10.1016/j.jep.2012.02.041 22414477

[B33] Ooi K. L. Tengku Muhammad T. S. Tan M. L. Sulaiman S. F. (2011). Cytotoxic, apoptotic and anti-alpha-glucosidase activities of 3,4-di-O-caffeoyl quinic acid, an antioxidant isolated from the polyphenolic-rich extract of *Elephantopus mollis* Kunth. *J. Ethnopharmacol.* 135 685–695. 10.1016/j.jep.2011.04.001 21497647

[B34] Ooi K. L. Tengku Muhammad T. S. Lam L. Y. Sulaiman S. F. (2012). Cytotoxic and apoptotic effects of Ethyl acetate extract of *Elephantopus mollis* Kunth. in human liver Carcinoma HepG2 cells through caspase-3 activation. *Integr. Cancer Ther.* 13 NP1–NP9. 10.1177/1534735411433203 22336595

[B35] Sabharwal S. S. Schumacker P. T. (2014). Mitochondrial ROS in cancer: initiators, amplifiers or an Achilles’ heel? *Nat. Rev. Cancer* 14 709–721. 10.1038/nrc3803 25342630 PMC4657553

[B36] Seong H. A. Manoharan R. Ha H. (2015). Coordinate activation of redox-dependent ASK1/TGF-beta signaling by a multi-protein complex (MPK38, ASK1, SMADs, ZPR9, and TRX) improves glucose and lipid metabolism in Mice. *Antioxid. Redox Signal.* 24 434–452. 10.1089/ars.2015.6325 26421442

[B37] Tabopda T. K. Ngoupayo J. Liu J. Shaiq Ali M. Khan S. N. Ngadjui B. T. (2008). Further cytotoxic sesquiterpene lactones from *Elephantopus mollis* KUNTH. *Chem. Pharm. Bull. (Tokyo)* 56 231–233. 10.1248/cpb.56.231 18239317

[B38] Takeuchi J. Hirota K. Itoh T. Shinkura R. Kitada K. Yodoi J. (2000). Thioredoxin inhibits tumor necrosis factor- or interleukin-1-induced NF-kappaB activation at a level upstream of NF-kappaB-inducing kinase. *Antioxid. Redox. Signal.* 2 83–92. 10.1089/ars.2000.2.1-83 11232604

[B39] Trachootham D. Alexandre J. Huang P. (2009). Targeting cancer cells by ROS-mediated mechanisms: a radical therapeutic approach? *Nat. Rev. Drug Discov.* 8 579–591. 10.1038/nrd2803 19478820

[B40] Uen Y. H. Liu D. Z. Weng M. S. Ho Y. S. Lin S. Y. (2007). NF-kappaB pathway is involved in griseofulvin-induced G2/M arrest and apoptosis in HL-60 cells. *J. Cell. Biochem.* 101 1165–1175. 10.1002/jcb.21240 17226769

[B41] Ueno H. Kajihara H. Nakamura H. Yodoi J. Nakamuro K. (2007). Contribution of thioredoxin reductase to T-cell mitogenesis and NF-kappaB DNA-binding promoted by selenite. *Antioxid. Redox Signal.* 9 115–121. 10.1089/ars.2007.9.115 17115890

[B42] Vardiman J. W. Thiele J. Arber D. A. Brunning R. D. Borowitz M. J. Porwit A. (2009). The 2008 revision of the World Health Organization (WHO) classification of myeloid neoplasms and acute leukemia: rationale and important changes. *Blood* 114 937–951. 10.1182/blood-2009-03-209262 19357394

[B43] Volpe G. Panuzzo C. Ulisciani S. Cilloni D. (2009). Imatinib resistance in CML. *Cancer Lett.* 274 1–9. 10.1016/j.canlet.2008.06.003 18653275

[B44] Wang S. Fang K. Dong G. Chen S. Liu N. Miao Z. (2015). Scaffold diversity inspired by the natural product evodiamine: discovery of highly potent and multitargeting antitumor agents. *J. Med. Chem.* 58 6678–6696. 10.1021/acs.jmedchem.5b00910 26226379

[B45] Wang Y. He Q. Y. Chiu J. F. (2014). Dioscin induced activation of p38 MAPK and JNK via mitochondrial pathway in HL-60 cell line. *Eur. J. Pharmacol.* 735 52–58. 10.1016/j.ejphar.2014.04.018 24755146

[B46] Yip N. C. Fombon I. S. Liu P. Brown S. Kannappan V. Armesilla A. L. (2011). Disulfiram modulated ROS-MAPK and NFkappaB pathways and targeted breast cancer cells with cancer stem cell-like properties. *Br. J. Cancer* 104 1564–1574. 10.1038/bjc.2011.126 21487404 PMC3101904

[B47] Zeisig B. B. Kulasekararaj A. G. Mufti G. J. So C. W. (2012). SnapShot: acute myeloid leukemia. *Cancer Cell* 22 698–698.e691. 10.1016/j.ccr.2012.10.017 23153541

[B48] Zhang Z. Y. Yu S. Q. Miao L. Y. Huang X. Y. Zhang X. P. Zhu Y. P. (2008). [Artesunate combined with vinorelbine plus cisplatin in treatment of advanced non-small cell lung cancer: a randomized controlled trial]. *Zhong Xi Yi Jie He Xue Bao* 6 134–138. 10.3736/jcim20080206 18241646

